# Perception of Loudness Is Influenced by Emotion

**DOI:** 10.1371/journal.pone.0038660

**Published:** 2012-06-07

**Authors:** Erkin Asutay, Daniel Västfjäll

**Affiliations:** 1 Civil and Environmental Engineering, Chalmers University of Technology, Gothenburg, Sweden; 2 Department of Behavioral Science and Learning, Linköping University, Linköping, Sweden; University of California, Davis, United States of Amercia

## Abstract

Loudness perception is thought to be a modular system that is unaffected by other brain systems. We tested the hypothesis that loudness perception can be influenced by negative affect using a conditioning paradigm, where some auditory stimuli were paired with aversive experiences while others were not. We found that the same auditory stimulus was reported as being louder, more negative and fear-inducing when it was conditioned with an aversive experience, compared to when it was used as a control stimulus. This result provides support for an important role of emotion in auditory perception.

## Introduction

Loudness perception is often described as a modular system where information is processed by dedicated auditory systems that do not communicate with other brain systems [1]. However, recent neuroscience research has shown that acoustic perception is affected by input from other modalities (e.g. visual processing), and that visual perception is affected by emotion processing. However, it remains unclear if, and how, auditory perception is influenced by emotion. In the present study, we examined if negative emotion can influence a basic sensory dimension: loudness perception.

The classical view of sensory organization, which contains segregated modality specific cortical streams that converge only at a later stage, is conflicted by growing multisensory integration research [2]. Two well-known examples of audio-visual interactions are the McGurk [3] and the ventriloquism [4] effects; where in the former, visual information in the form of lip-reading alters what is heard. In the latter case, presentation of a visual stimulus with a spatially conflicting auditory stimulus causes the perceived location of the sound to change. Furthermore, recent neurophysiological evidence indicates that brain areas that are considered as modality-specific could be affected by input from different modalities. For example, in a functional magnetic resonance imaging (fMRI) study, it was reported that lip-reading could affect auditory cortex [5]. Tones synchronized with a visual stimulus were shown to influence event-related-potentials (ERPs) in visual cortex [6]. Also, early modulation of auditory ERPs was found when listening to sentences that were presented with facial expressions of emotions [7]. These findings suggest that cross-modal interactions can influence primary sensory levels (for reviews see [8], [9]).

Furthermore, research has begun to explore the possibility that emotion processing may modulate low-level visual perception. Becker [10] reported that negative emotion leads to more efficient visual information search. Exposure to emotionally evocative faces has been linked to differential processing of low-level spatial information [11], [12], as well as a decreased field of view [13]. Furthermore, in binocular rivalry, where two images are presented to each eye and compete for dominance, emotional faces that are congruent with perceivers’ current emotional state increased their dominance [14], which shows that emotional state could influence the contents of consciousness. Barrett and Bar [15] claimed that the brains’ predictions made during visual object perception carry emotional value as a necessary part of visual experience. Based on neuroanatomical evidence, it was claimed that visual perception is informed by affect [15].

The influence of emotion on auditory perception has not received much attention. Some recent neurophysiological studies however suggest that emotion can influence early auditory processing. Wang and colleagues [16] found that negative emotion can affect auditory ERPs as early as 20 ms. Bröckelmann and colleagues [17], using an associative learning paradigm, found that early auditory processing is modulated by learned emotional meaning. Further, a recent study provides behavioral evidence that negative affect can influence loudness perception [18]. In this study, negative affect was induced by asking participants to write about a frightening experience from their past, and then participants rated loudness of a number of tones. Loudness ratings of participants in negative emotion condition were compared with a control group who were asked to write about their morning. As a result, participants in the negative emotion condition perceived the auditory stimuli louder compared to those that are in the control group.

Previous research has shown that auditory information readily and consistently induces emotional reactions (e.g. how physical intensity of a sound may influence the ensuing emotion by contributing to experienced arousal; [19]). Our goal here is to examine the inverse relationship: how do emotional reactions influence loudness perception?

The study presented here examines if negative emotion can influence loudness perception using a conditioning paradigm. The main difference between the present and aforementioned behavioral study [18] is that our goal is to attach emotional meaning to neutral auditory stimuli using low-level emotional learning and investigate whether learned emotional meaning can affect loudness perception. Drawing on work suggesting that one of the primary functions of the auditory system is to alert individuals about danger [20], and on neurophysiological [16], [17] and behavioral [18] evidence that negative emotion can influence auditory processing, we hypothesize that experienced negative emotion would increase perceived loudness. To test this hypothesis we used an evaluative conditioning paradigm in which some auditory stimuli were paired with an aversive experience, whereas others were not.

## Materials and Methods

### Participants, Materials, and Procedure

34 normal hearing individuals (9 females; mean age: 26.7±0.82) participated in the study. Participants were asked if they had a hearing problem. They gave their informed consent prior to the inclusion in the experiment and were compensated after the study. The experiment was conducted in accordance with the ethical standards in the 1964 Declaration of Helsinki, and was approved by the Västra Götaland regional ethics committee.

1/3 octave band wide noise with center frequencies 250 Hz, 500 Hz, 1 kHz and 2 kHz were used as auditory stimuli, which were 5-second long, sampled at 44.1 kHz, and had equal loudness at 5.5 sone [21]. Sounds were reproduced using two loudspeakers (Genelec 8030A) placed at 1.2 m height and 2 m distance from participants. The angle between the two loudspeakers was around 60 degrees from participants’ point of view.

Participants sat down on a chair, to which a startling vibration was applied via a powerful shaker (Monacor BR-25) that was attached on the backrest. Tactile stimulation, which was used as an aversive conditioning stimulus, was at 70 Hz and 300 ms long. The amplitude of the tactile stimulation was the same for every participant.

Moreover, a BIOPAC MP150 system equipped with a GSR100C amplifier was used in order to record participants’ electrodermal activity (EDA), which is a valid indicator of lower arousal range and is used as an index of conditioning in the majority of human conditioning studies [22]. Surface Ag/AgCl electrodes were attached on the medial phalanges of index and middle fingers of participants’ non-dominant hand.

**Figure 1:**
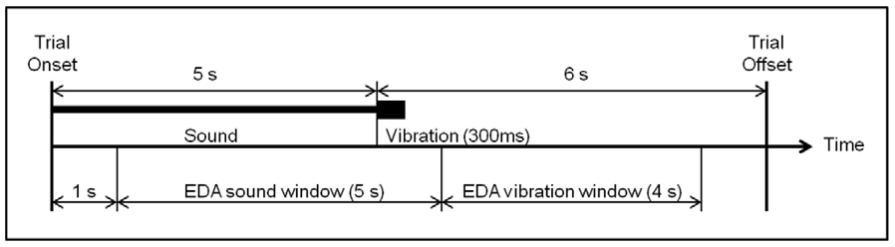
Timeline of a single conditioning trial. After the presentation of an auditory stimulus that was 5 seconds long tactile stimulus was presented. Between the onsets of two consecutive trials there were 11 seconds. Figure also shows the time windows in which EDA was scored.

The experiment was carried out in a dark, sound attenuated room, where participants completed all materials individually. First, participants completed a conditioning phase, in which two of the four sound stimuli (250 Hz and 2 kHz) were presented 6 times in a random order. After each repetition, one of the sounds (conditioned stimulus; CS+) was always followed by a moderately unpleasant tactile stimulation (vibration applied to the chair). The other sound served as a control stimulus (CS-), and was not paired with the tactile stimulation. Participants randomly assigned to one of the two groups: they either received 250 Hz or 2 kHz band noise as CS+. Between the onsets of two consecutive trials there were 11 seconds (Figure 1). In order to determine emotional impact of the aversive conditioning stimulus, we collected EDA responses to the tactile stimulation during the conditioning phase.

Immediately after the conditioning phase, participants listened to and rated loudness of each of the four sounds on a visual analog scale (VAS). Then, in a separate session, they rated how they felt when they heard each sound on 9-point scales (from 1 to 9) of valence (positive/negative content) and arousal (high/low arousal level) [23]. Also, in the same session participants, after listening to each sound, indicated how much fear they felt and how threatening they thought the sound was on separate VASs. Judgment sessions were self-paced. There was no resting period between judgment sessions, and participants did not perform any other task in these sessions. The order of the two judgment sessions was alternated (i.e. half of the participants rated loudness of the stimuli first and the other half rated their emotions first). The subjective measures of emotion were introduced, to investigate if the emotional reactions to the auditory stimuli were modulated by the conditioning manipulation.

### Data Analysis

During the conditioning phase, EDA was scored for each repetition of the auditory stimuli within a time window, which started 1 second after the onset of the stimulus and lasted until 1 second after the offset. Further, EDA responses to the tactile stimulation were scored within a 4-second time window which started 1 second after the onset of the tactile stimulation and ended 1 second before the onset of the following trial. Within the specified time windows, the signals were band-pass filtered between 0.2 and 3 Hz. High pass filtering is applied in order to filter out the tonic component of EDA, and low pass filtering was done in order to get rid of high frequency noise. The resulting signal was full-wave rectified and integrated to a single value. Finally, log transformation was done before the data was standardized for each participant [24]. Scoring EDA in this manner is proposed due to the fact that it provides freedom to the experimenter to select inter-stimulus-intervals. Finger and Murphy [24] suggested a band-pass filtering between 0.5 and 2 Hz. In our study, both their suggestion and the filter applied (between 0.2 and 3 Hz) yielded the same results. EDA data was analyzed in a 2 (conditioning group) × 2 (sound) × 6 (repetitions) repeated-measures analysis of variance (ANOVA).

All judgments made on VASs (i.e. loudness, fear, threat) were standardized for each participant. After initial exploration of the data, it was found that valence ratings were positively skewed (Skewness = .42, SE of skewness = .21). This might be due to the fact that the auditory stimuli were perceived either negative or neutral. Only 17 of the 136 collected valence ratings were above 5. In order to reduce skewness in valence judgments a square root transformation was applied (which reduced skewness to −.13 with the same SE).

Judgment data were analyzed using 2 (conditioning group) × 4 (sound) repeated-measures ANOVA. We expected an interaction of the factors, and in order to make focused comparisons we employed contrast analysis [25]. The largest differences between groups were expected for 250 Hz and 2 kHz band noise in opposite direction. Hence, we searched for a linear contrast of the interaction along the center frequency of auditory stimuli. The interaction effect of the factors (conditioning group and sound) has 3 degrees of freedom, and the appropriate error term for the interaction has 96 degrees of freedom. However, the linear trend of the interaction has only one degree of freedom. Also, in contrast analysis one can construct a specific error term for each contrast [25]. In practice, this is done by separating the sum of squares of the error term into independent parts for each specific contrast. In this case, the interaction itself has 3 degrees of freedom; hence, a specific error term for a single contrast has 32 degrees of freedom (i.e. 96/3). Therefore, F-statistics for the linear trend of the interaction for the judgment data is F(1,32). The weights assigned to judgment data for the group that received 250 Hz band noise as CS+ were 0.671, 0.224, −0.224, and −0.671 for the auditory stimuli 250 Hz, 500 Hz, 1 kHz and 2 kHz, respectively. For the other group, the weights were −0.671, −0.224, 0.224, and 0.671. These weights were assigned by the SPSS statistical software in a way that sum of squares of weights for each group would be one.

## Results

**Figure 2:**
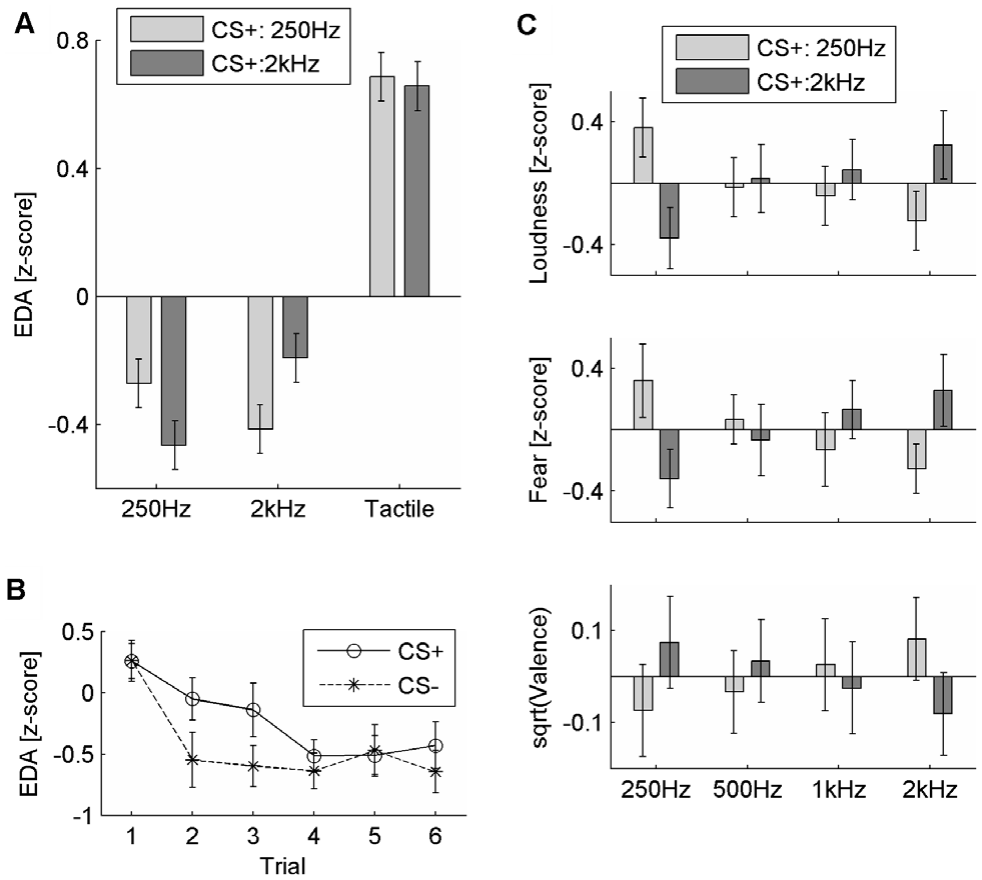
Results. (**A**) Mean EDA induced by auditory and tactile stimuli during conditioning phase shown for the two conditioning groups (CS+: 250 Hz vs. CS+: 2 kHz). SE is indicated. (**B**) Average EDA induced by CS+ and CS- in the conditioning phase (the two conditioning groups combined) at different trials. Standard errors of the means are indicated. (**C**) Interaction effect of conditioning group and sound on loudness (top), fear (middle) and valence (bottom) judgments. Main effects and grand means are removed. SE is indicated.

First, the EDA responses to auditory and tactile stimuli during the conditioning phase were investigated (Figure 2A). Tactile stimulation induced significantly higher EDA compared to auditory stimuli (F(1,32) = 122.46, p<.001, η^2^ = .79; and F(1,32) = 109.66, p<.001, η^2^ = .77 for 250 Hz and 2 kHz band noise, respectively). Further, consistent with previous conditioning literature [26], a significant conditioning group and sound interaction indicated that participants in both groups had higher EDA when they heard CS+ compared to CS- (F(1,32) = 4.82, p<.05, η^2^ = .13, Figure 2A, 2B). These findings suggest that tactile stimulation was emotionally arousing on its own, and that we successfully altered the emotion associated with the auditory stimuli.

In a critical test of our hypothesis, a significant linear contrast of conditioning group and sound interaction (F(1,32) = 5.50, p<.05, η^2^ = .15; Figure 2C) confirmed that CS+ were perceived as louder than CS- in both groups. Also, significant linear contrasts of group and sound interactions were found for both fear (F(1,32) = 4.86, p<.05, η^2^ = .13) and valence (F(1,32) = 4.22, p<.05, η^2^ = .12) judgments where CS+ were rated as more negative and fear-inducing (Figure 2C). Table 1 presents mean loudness, valence and fear ratings for each auditory stimulus by the two conditioning groups.

Further, the dimensional correlation between EDA responses during the conditioning phase and loudness judgments was investigated. A positive correlation (r = .22, p = .03, one-tailed, N = 68) was found between participants’ loudness judgments and their average EDA response during the conditioning phase to each stimulus. The dimensional correlation between the loudness judgments and average EDA responses during the second and third trials of the conditioning phase, where largest differences occurred between CS+ and CS- (Figure 2B), was significant (r = .33, p<.01, N = 68). Further, the dimensional correlation between the loudness judgments and the subjective measures of emotion was investigated for CS+ and CS-. It was found that loudness judgments positively correlated to arousal (r = .21, p = .09, N = 68), fear (r = .25, p<.05, N = 68) and threat (r = .30, p<.05, N = 68) judgments, whereas it negatively correlated to valence judgments (r = −.25, p<.01, N = 68).

**Table 1:** Mean valence, fear and loudness ratings for the auditory stimuli according to the two conditioning groups (CS+: 250 Hz vs. CS+: 2 kHz).

		Valence: M (SE)		Fear: M (SE)			Loudness: M (SE)	
	CS+:250 Hz		CS+:2 kHz	CS+:250 Hz	CS+:2 kHz	CS+:250 Hz		CS+:2 kHz
250 Hz	3.82 (.44)		3.71 (.36)	.358 (.231)	−.276 (.245)	.124 (.178)		−.598 (.207)
500 Hz	3.65 (.39)		3.35 (.34)	.124 (.203)	−.009 (.177)	.306 (.230)		.362 (.162)
1 kHz	4.12 (.4)		3.35 (.32)	−.064 (.177)	.195 (.140)	−.123 (.215)		.049 (.167)
2 kHz	3.76 (.28)		2.76 (.35)	−.418 (.202)	.091 (.262)	−.308 (.206)		.187 (.239)

Fear and loudness ratings are z-scores. Valence ratings are on a scale from 1 to 9.

## Discussion

The present study set out to investigate whether experienced negative emotion could influence loudness judgments. In order to test this hypothesis, we employed an evaluative conditioning paradigm where some auditory stimuli were paired with an aversive experience. We used meaningless auditory stimuli that were separated by their spectral content, and each had the same loudness. Our goal was to use emotion conditioning to assign negative emotional meaning to initially emotionally-neutral sounds. Importantly, we predicted that conditioning would change not only the emotional reaction to sounds, but also the perception of them (i.e. increased loudness).

Firstly, the EDA results suggested that the conditioning stimulus was emotionally arousing. Further, during the conditioning phase, CS+ induced higher EDA compared to CS- regardless of the actual sounds that were used as CS+ and CS-. Even though the conditioning worked as intended, there seemed to be a habituation effect during the conditioning phase over trials 4–6 (Figure 2B). We are not certain about the reason for this; however, it might be because the auditory stimuli were not fear-relevant. Öhman and Mineka [22] discussed that fear-relevant stimuli (e.g. picture of a snake) were more effective as conditioned stimuli and more resistant to extinction compared to fear-irrelevant stimuli (e.g. picture of a house). Regardless of the fact that there seemed to be a habituation effect, our results suggest that CS+ gained emotional value due to consistent pairing with the aversive experience, which is also supported by subjective measures. Statistical analyses confirmed that CS+ was regarded as more fear-inducing and negative compared to CS- (Figure 2C). Finally, we found differences in loudness judgments and their correlation with the EDA responses during the conditioning phase indicating that induced negative emotion by auditory stimuli can influence loudness perception. Siegel and Stefanucci [18] addressed the same issue and found similar results using a different paradigm. They induced negative affect using an incidental mood induction manipulation (based on recollections of memories; a high-level cognitive manipulation) and collected loudness ratings of neutral stimuli. Our study lends further evidence to negative emotion and auditory perception interactions; we found that loudness perception can be influenced by emotional meaning of the auditory stimulus itself (integral emotion) and that this can occur through low-level affective learning.

Emotional stimuli have been argued to receive prioritized sensory processing as a possible survival-related mechanism [27]. This might be one of the reasons why negative emotion can influence loudness perception. Emotionally salient auditory stimuli might cause increased sensitivity to loudness, so that a quick response could be generated when necessary. This explanation seems reasonable when one considers the role of the auditory system as a warning system that constantly scans the immediate environment surrounding the organism and informs changes and potential dangers in it [20]. This reasoning is in line with Mineka and Öhman’s finding [28] that fear-relevant stimuli are selective and impenetrable to cognitive conscious control, and that it has an adaptive function. Nevertheless, further research should address this issue. Future work should also focus on the influence of emotion on different aspects of auditory processing very much like the studies in the visual domain [10]–[15].

We can speculate about the neural basis for our findings. For instance, previous research has shown that the amygdala, which influences visual processing [29] and perception [30], is also involved in conditioning and has projections to auditory thalamus and auditory cortex [31]. Emotion processing may influence auditory perception in a similar manner. Furthermore, associative learning seems to induce short-term plasticity in human auditory cortex, which can acquire and retain specific information about the behavioral significance of auditory stimuli [32]. Bröckelmann and colleagues [17] discussed that modulation of early auditory event-related magnetic fields due to learned emotional meaning of the stimuli might be related to abovementioned learning induced plasticity in auditory cortex and influence of top-down attentional filter functions [33].

In sum, regardless of exact neural basis and mechanisms, our results show that the same sound was reported as more fear-inducing and negative, and perceived as louder when it was conditioned with an emotionally arousing event, compared to when it was used as a control stimulus. This suggests that emotion is an important mechanism for auditory perception. Thus, research on auditory perception must start to acknowledge the important role of emotion in sensation and perception.
